# Implementing a hospital-wide protocol for *Staphylococcus aureus* bacteremia

**DOI:** 10.1007/s10096-018-3284-9

**Published:** 2018-05-31

**Authors:** K. Bolhuis, L. J. Bakker, J. T. Keijer, P. J. de Vries

**Affiliations:** 1 Department of Internal Medicine, Academic Medical Center, Meijbergdreef 9, 1105 AZ Amsterdam, The Netherlands; 2 Department of Medical Microbiology, Tergooi Hospital, Van Riebeeckweg 212, 1213 XZ Hilversum, The Netherlands; 3 Department of Cardiology, Tergooi Hospital, Van Riebeeckweg 212, 1213 XZ Hilversum, The Netherlands; 4 Department of Internal Medicine, Tergooi Hospital, Van Riebeeckweg 212, 1213 XZ Hilversum, The Netherlands

**Keywords:** *Staphylococcus aureus*, Bacteraemia, Treatment, Echocardiography, Infective endocarditis

## Abstract

*Staphylococcus aureus* bacteraemia (SAB) is associated with high-mortality and complication rates. A multidisciplinary approach is needed to predict, detect and treat complications. In this pre- and post-intervention study, we investigated the effects of a hospital-wide protocol for diagnosis, classification and treatment of SAB. It was hypothesized that complications and endocarditis would be better identified and treated. Medical records of SAB patients admitted in 2011 and 2012 (pre) were analysed. In 2013, a protocol, describing risk factors, diagnostic classification and recommended treatment, was implemented. In 2014 and 2015 (post), SAB patients were followed prospectively. Transthoracic (TTE) or transoesophageal cardiac ultrasound (TEE) was chosen following a decision tree. A resident internal medicine acted as contact person. Pre-intervention, 98 patients were eligible for analysis compared to 85 patients post-intervention. Age and number of risk factors were slightly higher post-intervention; other baseline characteristics were similar. Most SAB-patients were classified as complicated (89 and 82% pre- and post-intervention, respectively). Follow-up blood cultures drawn within 2 days after initiating treatment increased from 51 to 85%. Cardiac ultrasounds increased from 44 to 83% for TTE and 13 to 24% for TEE. Endocarditis was more frequently diagnosed (4 vs. 12%). Additionally, duration of antibiotic therapy increased. The 3-month mortality did not change significantly (33% pre-intervention vs. 35% post-intervention; *p* > 0.05). Introduction of a hospital-wide protocol for SAB management increased standard of care, created awareness among clinicians to properly classify SAB, search for endocarditis and adapt duration of antibiotic treatment. Mortality did not decrease.

## Introduction

*Staphylococcus aureus* bacteraemia (SAB) is associated with a long hospital stay, a high morbidity and high 30-day mortality numbers ranging from 10 to 30% [1, 2]. The mortality is associated with the development of serious complications such as metastatic abscesses, vertebral osteomyelitis and acutely life-threatening complications such as endocarditis, intracardiac abscesses and valve perforation [3]. Important in the management of SAB is to correctly classify SAB as either uncomplicated or complicated and to detect the complications as quickly as possible. Risk factors for a complicated course include community acquisition, persistent positive blood cultures after 48–72 h, persistent fever after 72 h, diabetes mellitus, purulent thrombophlebitis, presence of prosthetic devices and immunosuppressive therapy [4–6]. In addition, repetitive follow-up blood cultures and echocardiography are recommended for patients with risk factors to detect (imminent) complications.

The antibiotic treatment of SAB aims to prevent deterioration of disease and the development of complications. The duration of treatment remains somewhat controversial, but current guidelines regarding management of SAB patients recommend at least 14 days of antimicrobial therapy for a simple bacteraemia with low risk of complications. For bacteraemia with risk factors associated with a complicated course due to metastatic infections or endocarditis, at least 28 days of antimicrobial therapy is recommended [4, 7–10]. In contrast to many other blood stream infections, the instalment of adequate antibiotic treatment is not enough to warrant a favourable outcome.

Although SAB is among the deadliest bloodstream infections, previous studies showed that potentially complicated infections are not well recognized by physicians resulting in non-adherence to standard of care therapy in 10 to 41% of SAB cases [11–13]. Detection of complications remains a challenge because the clinical symptoms and findings are non-specific [14, 15]. Despite the identification of several risk factors for developing these complications, mortality rates have only improved marginally [16].

Non-adherence to standard of care is most often the result of inadequate duration of antibiotic treatment, lack of follow-up blood cultures, or omitting echocardiography [12–14]. In particular, transoesophageal echocardiography (TEE) is often not performed due to comorbidity, limited acceptance by patients or treating physicians [12], even though other authors showed that performing routinely TEE results in a high incidence of the most feared complication: infective endocarditis [17].

Multiple studies have shown an increase in adherence to standard of care by routine infectious disease (ID) consultation and some found that this adherence seemed to preface a significant lower short-term mortality rate (or variable effect on mortality rates) [14, 18–21]. Almost all those studies were performed in large academic, tertiary hospitals, and it remains to be confirmed whether this can be generalized to non-academic, regional or smaller hospitals.

To improve quality of care, we conducted an intervention study, a multidisciplinary approach to predict, detect and treat complications of SAB and adapt the treatment to the risk profiles. The primary goal of this study was to implement a clear, structured, safe and univocal hospital-wide protocol regarding the management and treatment of patients with SAB and to evaluate the effects of this protocol in a regional teaching hospital.

## Materials and methods

### Study design

This pre- and post-intervention retrospective study was performed in a 550-bed regional teaching hospital in the centre of the Netherlands. In cooperation with a cardiologist, medical microbiologist and ID specialist, a protocol was designed that describes the risk factors of complicated SAB and includes a flow chart for diagnostic classification and recommendations for therapy according to the current guidelines. Pre-intervention data were collected retrospectively from the hospital records. The post-intervention data were collected prospectively.

All events of *S. aureus* bacteraemia in patients > 16 years were analysed during the study period. A SAB > 12 weeks after cessation of antimicrobial therapy for a previous SAB was considered to be a distinct event and was included into the analysis. Because the study was designed to evaluate the effect of implementation of a protocol, exclusion criteria included contaminated blood culture specimen or any of the following within 3 days after collection of blood culture sample: death, withdrawing of therapy or transfer to another facility. Pregnancy was an exclusion criterion as well.

### Pre-intervention group

The pre-intervention group cohort was studied in retrospect. All records of hospitalized patients with blood cultures positive for *S. aureus* between 1 January 2011 and 31 December 2012 were retrieved. The date of the first sample taken was considered the date of diagnosis. This group was called the pre-intervention group.

### Intervention protocol

In 2013, accompanying the introduction of a hospital-wide protocol, awareness was created by oral and visual presentations to all medical staff. The potential complications of SAB and the added value of TEE were emphasized in these educational sessions. All cardiologists were made aware of the protocol. The protocol was implemented in all medical and surgical wards and was designed to function as a simple guideline for physicians by classifying patients to groups with uncomplicated SAB or with potentially complicated SAB. Within complicated SAB, possible endocarditis was identified as a separate group.

Patients with SAB were reported by the laboratory of microbiology as soon as a blood culture became positive for *S. aureus*. The attending physician was informed about this SAB and referred to the protocol. According to the protocol SAB was considered complicated when at least one of the following risk factors was present: community acquisition, diabetes mellitus, persistent positive blood cultures after 48/72 h of antibiotic therapy, prosthetic material in situ, persistent fever after 72 h of therapy and metastatic infections. These risk factors are known to be associated with a high risk for a complicated course [4–6].

**Fig. 1:**
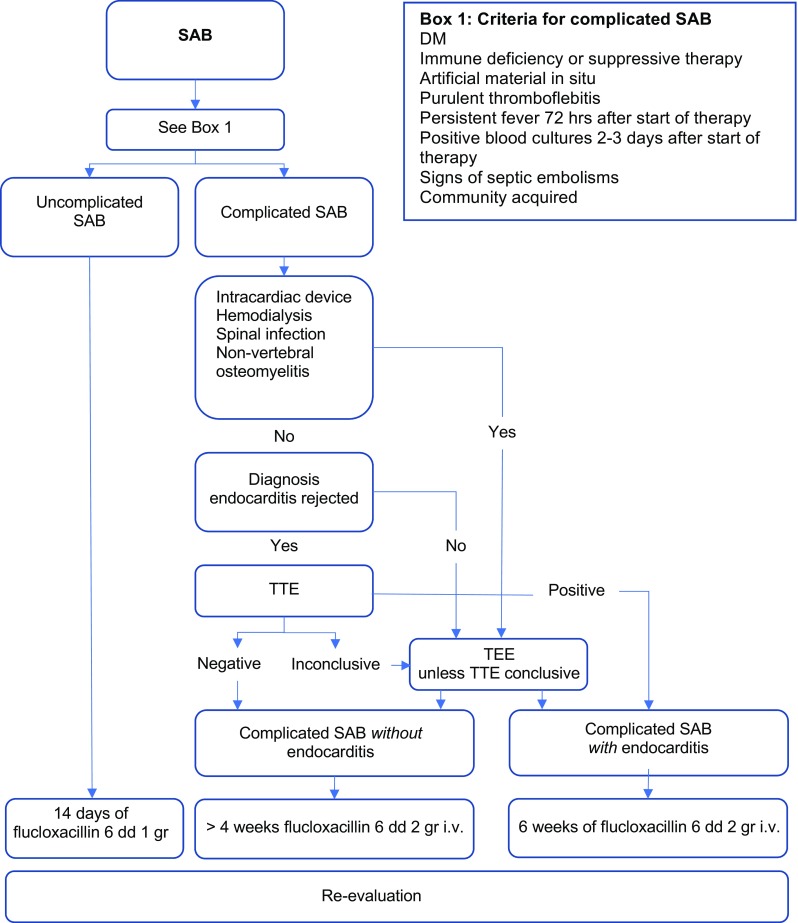
Protocol for diagnosis, classification and treatment of SAB. SAB, *Staphylococcus aureus* bacteraemia; TTE, transthoracic echocardiography; TEE, transoesophageal echocardiography

**Fig. 2:**
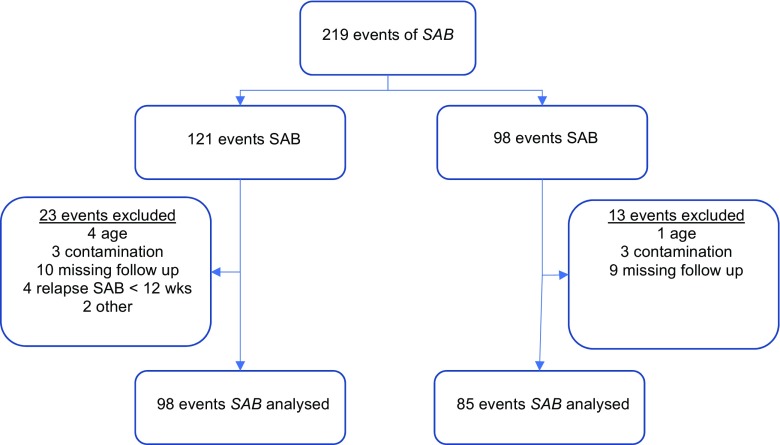
Flow diagram of events included in the study. SAB, *Staphylococcus aureus* bacteraemia

Before and during the intervention, an ID specialist (PdV) was working in the hospital. The introduction of the protocol introduced a closer involvement of the ID specialist and the dedicated resident internal medicine (KB) to the patient care but did not include bedside visits by them. The flow chart guided the treating physicians to score these risk factors and classify the patients in complicated versus uncomplicated (Fig. 1). Flucloxacillin is the drug of first choice in the Netherlands for patients with SAB. According to the flowchart, uncomplicated SAB was treated with flucloxacillin intravenously for 14 days. For complicated SAB, the flowchart advises to assess the modified Duke criteria for endocarditis and to perform a transthoracic echocardiography (TTE) for all patients. When the echocardiographic results were uncertain or inconclusive, a transoesophageal echocardiography (TEE) was suggested. When indicated, echocardiography was conducted by the attending cardiologist, usually the same day. Depending on the outcome of the echocardiography, at least 4 weeks of IV flucloxacillin therapy was recommended for these complicated SAB-patients. If infective endocarditis was confirmed, the patient was treated according to national guidelines which includes 6 weeks of IV flucloxacillin and 3 to 5 days of low-dose gentamicin, with or without rifampicin (Fig. 2).

### Post-intervention group

The post-intervention group consisted of all SAB episodes of admitted patients between 1 January 2014 and 31 December 2015. These patients were followed prospectively with respect to the routinely collected data. Consequently, in this study, prospectively collected data were compared to data of recent historic cohort. In the post-intervention period, a dedicated resident internal medicine, advising microbiologist and ID specialist actively recommended the attending physician to consult and follow the protocol, which is available on the hospital’s intranet pages. The management of individual patients including actually performing echocardiography remained the responsibility of the treating physician.

The study was approved by the hospital’s scientific review committee responsible for improving the quality of care. This study did not collect experimental data, and since the data collection itself is part of the routine quality of care assessment system, no additional ethical clearance was required.

### Patient follow-up

Patients were followed until death or 6 months after diagnosis, whichever of the events occurred first. The medical records were analysed, and data were collected by a single investigator.

### Data collection

Medical records were analysed for patient characteristics, medical history, underlying diseases, source of infection, presence of prosthetic devices and other known risk factors. Information about choice, dosage and duration of antimicrobial therapy was recorded; number of follow-up blood cultures and number and results of echocardiograms were also included. All SAB-related complications, including the occurrence of endocarditis, relapse of infection and mortality, were recorded.

Hospital-acquired bacteraemia was defined by isolation of *S. aureus* from a blood culture obtained > 72 h after hospital admission. Purulent thrombophlebitis was diagnosed, based on clinical symptoms and signs.

When the source of infection was, for example, an indwelling peripheral intravenous catheter, a pacemaker or a central venous catheter, this was defined as a health care-related infection, and removal of these intravenous devices was scored.

Adequate antibiotic treatment was defined as the initiation of parenteral administration of antibiotics with documented activity against the isolated *S. aureus* strain, with adequate dosage and duration of therapy (14 days for uncomplicated and at least 28 days for complicated SAB).

Infective endocarditis was scored based on echocardiography, modified Duke criteria [22], strongly suggestive clinical findings, PET-CT or endocarditis found at autopsy.

Relapse of infection was defined as recurrent isolation of *S. aureus* in blood cultures within 12 weeks after cessation of antimicrobial therapy.

The most important post-discharge outcomes were relapse of SAB and mortality. In case of relapse, it was expected that most if not all patients would be referred and admitted to the hospital due to the complicated natural course of SAB and reasoning that SAB is not self-limiting in case no intravenous antimicrobial treatment is offered to the patient. Following this theory, possible relapses were scored by analysing the medical records.

Mortality outside the hospital was collected by medical records as well. Tergooi hospital receives and records the information of deaths of all patients, even if they do not die in the hospital.

### Primary endpoint output

Adherence to standards of care was scored by three indicators: (1) obtaining follow-up blood cultures 2 to 4 days after initiation of (adequate) therapy; (2) administration of adequate therapy scored by the use of appropriate antibiotics, optimal parenteral treatment dosage and duration; and (3) performing echocardiography.

### Data analysis

Data were initially entered in Microsoft Excel and analysed in SPSS (v. 23, SPSS Statistics for Windows, Version 23.0. Released 2015. IBM Corp. Armonk, NY).

Parametric tests were used for comparison of normally distributed variables. Non-parametric tests were used otherwise. Categorical variables were analysed with chi-square tests. Statistically significant difference was accepted when *p* < 0.05.

#### Data availability

The datasets generated during and/or analysed during the current study are available from the corresponding author on reasonable request.

## Results

A number of 219 patients was diagnosed with at least one blood culture specimen positive for *S. aureus*. Thirty-six patients were excluded, six due to contaminated blood cultures, five patients were < 16 years old, four because of a relapse within 12 weeks and 19 were lost to follow-up within 3 days because of death, withdrawing of therapy or translocation to another facility. A total of 98 patients remained eligible for analysis in the pre-intervention group and 85 patients in the post-intervention group (Fig. 1).

Demographic characteristics are shown in Table 1. Two patients, both in the pre-intervention group, suffered from more than one episode of *S. aureus* bacteraemia (defined as a new episode of SAB > 12 weeks post-therapy of the previous episode of SAB). In the first patient, the episodes were separated by 9 months, and in the second patient, the episodes were separated by 3 years.

**Table 1:** Patient demographic characteristics. *Staphylococcus aureus* bacteraemia before (2011–2012) and after a protocol adherence intervention (2014–2015)

Variable	Pre-intervention (*n* = 98)	Post-intervention group (*n* = 85)	*p* value
Age, median years (min, max)	69, 65 (19, 94)	76.49 (35, 98)	*0.016*
Female sex	47 (48%)	35 (41%)	*0.44*
Classification SAB			
Uncomplicated SAB	11 (11%)	15 (18%)	*0.303*
Complicated SAB	87 (89%)	70 (82%)	
Risk factors			
Community acquisition	63 (64%)	55 (65%)	
Diabetes mellitus	28 (29%)	26 (31%)	
Positive follow-up blood cultures 48/72 h	13 (13%)	21 (25%)	
Prosthetic material	31 (32%)	29 (34%)	
Persistent fever after 72 h	4 (4%)	9 (11%)	
Catheter-related and in situ	3 (3%)	2 (2%)	
Immunocompromised	12 (12%)	9 (11%)	
Metastatic infections	23 (23%)	23 (27%)	
Purulent thrombophlebitis	11 (11%)	0 (0%)	
Malignancy	18 (18%)	10 (12%)	
Alcohol	10 (10%)	7 (8%)	
Haemodialysis	3 (3%)	1 (1%)	
No. of risk factors			
0	11 (11%)	15 (18%)	*0.590*
1	27 (28%)	16 (19%)	
2	31 (32%)	28 (33%)	
3	22 (22%)	16 (19%)	
4	5 (5%)	6 (7%)	
5	3 (3%)	4 (5%)	
Source of infection			
Health care-related	50 (51%)	37 (44%)	
Surgery	11 (22%)	6 (16%)	
Urinary tract/CAD	6 (12%)	8 (22%)	
CVC/PAC	11 (22%)	5 (14%)	
Peripheral catheter	12 (24%)	11 (30%)	
Unknown source	26 (52%)	21 (57%)	

Patient demographics. *SAB*, *Staphylococcus aureus* bacteraemia; *CAD*, urine catheter; *CVC*, central venous catheter; *PAC*, port a cath; NS, not significant. A *p* value < 0.05 is considered statistically significant

The median age was higher in the post-intervention group compared to the pre-intervention group, and malignancy was more frequent in the pre-intervention group, but other baseline characteristics were similar. Most SAB patients were classified as complicated (89 and 82% pre- and post-intervention, respectively).

The adequacy of care for SAB increased during the intervention (Table 2). Firstly, the frequency of drawing follow-up blood cultures 48–72 h after initiating treatment significantly increased, resulting in the identification of more patients with positive blood cultures during follow-up (15 vs. 25%). In both groups, the proportion of positive blood cultures remained the same (29 vs. 29%). Secondly, the mean duration of antibiotic therapy significantly increased (17.3 vs. 23.0 days*)*, resulting in more patients receiving adequate antimicrobial therapy (39 vs. 71%). And, thirdly, among patients with complicated SAB, more TTEs were performed (44 to 83%), and the number of TEEs increased from 13 to 24%. Consequently, endocarditis was detected more frequently (12%) during the intervention period than before (4%). The increase of TEE did not decrease the proportion of positive TEEs (31 vs. 42%). This underlines the urgency to perform a TEE.

**Table 2:** Quality of care indicators

Diagnostic and therapeutic management of *Staphylococcus aureus* bacteraemia, before (2011–2012) and after a protocol adherence intervention (2014–2015)			
Variable	Pre-intervention (*n* = 98)	Post-intervention (*n* = 85)	*p* value
Diagnostic workup			
Echocardiography performed in complicated SAB patients			
TTE	38 (44%)	58 (83%)	*< 0.001*
TEE	11 (13%)	17 (24%)	*0.09*
2nd blood cultures obtained after 48–72 h	50 (51%)	72 (85%)	*< 0.001*
Adequate antibiotic therapy			
Yes	38 (39%)	60 (71%)	*< 0.001*
No	59 (58%)	24 (28%)	
Not started	4	0	
Inadequate length	53	24	
Inadequate dose	3	0	
Unknown	1 (1%)	1 (1%)	
Intravenous antibiotic treatment			
< 14 days	32 (33%)	8 (9%)	
≥ 14 days	44 (45%)	58 (68%)	
Lost to FU < 14 days	18 (18%)	19 (22%)	
Death/withdrawing therapy	15	16	
Transfer	2	2	
Unknown	1	1	
Adequate AB not started	4 (4%)	0 (0%)	
Antibiotics stopped due to death or withdrawing therapy			
Adequate AB not started	4	0	
Yes	23	21	
No	65	57	
Transfer	5	5	
Unknown	1	2	
Treatment duration (mean)^a^	17.3 (*n* = 65)	23.0 (*n* = 57)	*0.014*
Uncomplicated SAB (mean/days)	10.3 (*n* = 7)	13.8 (*n* = 15)	
Complicated SAB (days, mean)	18.2 (*n* = 58)	26.3 (*n* = 42)	
Length of hospital stay^c^ (mean, days)	27.5	22.4	NS

*SAB*, *Staphylococcus aureus* bacteraemia; *TTE*, transthoracic echocardiography; *TEE*, transoesophageal echocardiography; *FU*, follow-up; *AB* antibiotic treatment; NS, not significant. A *p* value < 0.05 is considered statistically significant.

^a^Mean treatment duration. Patients were excluded when adequate antibiotics were not started or if antibiotic therapy was stopped due to death, withdrawing therapy or transfer to a different health care clinic

^b^Echocardiography in complicated SAB patients

^c^Length of hospital stay was calculated from date positive blood culture was taken till day of discharge. Patients transferred to different hospital were excluded

More infectious complications were detected in the post-intervention group (39 vs. 45%), and the mean duration of hospital stay, counting from the date of the first positive blood culture, decreased from 27 days before intervention to 23 days post-intervention. In addition, lower number of relapses within 12 weeks were reported (5 vs. 2%) (Table 3).

**Table 3:** Secondary outcomes

Variable	Pre-intervention (*n* = 98)	Post-intervention group (*n* = 85)
Infectious complications	38 (39%)	38 (45%)
Endocarditis	4 (4%)	10 (12%)
Spondylodiscitis	8 (8%)	5 (6%)
Abscesses	10 (10%)	13 (15%)
Septic arthritis	3 (3%)	6 (7%)
Prosthetic joint infection	5 (5%)	7 (8%)
Endovascular infection	2 (2%)	2 (2%)
Relapse within 3 months	5 (5%)	2 (2%)
Cerebral septic embolism	4 (4%)	4 (5%)
Mortality		
30-day mortality	25 (26%)	22 (26%)
Uncomplicated	3	1
Complicated	22	21
Unknown/lost to FU	2	1
90-day mortality	32 (33%)	30 (35%)
Uncomplicated	4	3
Complicated	28	27
Unknown/lost to FU	3	1

Secondary outcome: complications. *FU*, follow-up

The 4- and 12-week mortality did not change significantly (26 vs. 26% and 33 vs. 35% in the pre-intervention vs. post-intervention group; *p* > 0.05).

## Discussion

This study shows that introducing a hospital-wide protocol for management of SAB in a general teaching hospital improved the standard of care: The number of follow-up blood cultures significantly increased by two thirds, antimicrobial therapy became significantly more adequate and the number of TTE and TEE almost doubled post-intervention.

It created awareness among clinicians to properly classify SAB, search for endocarditis, choose the right antibiotic regimen and adapt duration of antibiotic treatment. However, it did not improve survival.

One of the strong points of this study is its real-life setting. A small intervention such as the introduction of this protocol creates knowledge about the treatment approach involving patients with SAB among a wide range of medical specialists and thereby directly improves standard of care. The introduction of protocols and local guidelines probably belong to the most common and feasible interventions in hospitals. The situation in our hospital reflects the situation of many hospitals, and this intervention can easily be implemented in many other hospitals. In our knowledge, this study is among the largest studies performed in the setting of a general hospital [23].

In this study, the motivations to change practices were not studied in depth. However, we encountered some expressions of inertia towards change. These were mainly the lack of knowledge and sense of urgency with respect to the poor prognosis of SAB and its treacherous presentation of complications. Another explanation for the relatively persistent low number of TEE might be the fact that, in exceptional cases, the protocol permitted clinicians to forego TEE in patients without any clinical signs of endocarditis, with excellent acoustic window on TTE and no vegetations or valvular regurgitation (Fig. 1). We did not encounter any expressions of mistrust by the attending physicians towards the resident or ID specialist.

There was often reluctance to conduct transoesophageal echocardiography because it was considered too invasive for ill and elderly patients and falsely relying on the acuity of the cardiac valve images during transthoracic echocardiography. PET-CT was available already before the intervention but located in another hospital with which it was shared. After the study, a PET-CT facility was installed at the location of the study. It is nowadays increasingly used to detect complications of SAB.

The contribution of purulent phlebitis with bacteraemia decreased (Table 1) substantially. This reduced the total number of complicated SAB while the proportional detection of persistent bacteraemia increased.

Previous studies showed the beneficial effects of implementation of hospital-wide diagnostic and therapeutic guidelines and of the standard involvement of an infectious disease consultant on adherence to standard of care [13, 18]. Borde et al. implemented a standardized bundle approach to the treatment of *S. aureus* bacteraemia which resulted in an increase of conducting TEE by 42% and collecting follow-up blood cultures by 49% [18]. Appropriate therapy within 72 h of detection of SAB increased by 81%.

In our study, we also found significant effects on quality of care indicators; the adequacy of dosing and duration of antimicrobial treatment and on documentation of infectious complications. An interesting phenomenon was found by analysing the duration of hospital stay before and after intervention. Despite a longer mean antimicrobial treatment, the mean duration of hospital stay decreased after protocol implementation. We hypothesize that, greater awareness among clinicians, more patients receiving optimal treatment and the use of more diagnostic tools to find infectious complications prevents serious complications during hospital stay and leads to faster hospital discharge.

The year 2013 was not analysed but used to implement the SAB protocol. After proper implementation, we started to collect data prospectively from 2014 onwards. The long study period, 5 years, has the advantage of excluding season effects. Although this may also introduce effects of changing health care and outcomes over time, we are not aware of important structural changes regarding the medical care in this hospital during this period. The only effect was a reduction of purulent phlebitis. The extra attention and instructions about SAB and its causes might have improved awareness about the risks and better measures to prevent infections due to peripheral catheters.

Retrospective studies may be subject to different forms of bias, but in this study, we captured all patients with documented SAB in a similar manner before (the retrospective part) and after intervention. This makes bias less likely. This study was not blinded but since data collection was similar before intervention as after, we do not consider this a probable source of bias. Follow-up was incomplete for several patients who were discharged from the hospital for palliative/terminal home care or who were transferred to other institutions.

We saw a threefold increase of the detection of endocarditis after intervention. Still, most patients with complicated SAB did not receive a TEE.

Endocarditis is among the most feared complications of SAB with a reported incidence of 25–29% and a 1-year mortality rate up to 43% [3, 15, 24]. This complication is difficult to diagnose since the initial clinical symptoms and signs are insensitive and non-specific [14, 15]. Echocardiography is recommended as primary tool to diagnose endocarditis but clinicians should be aware about its limitations. TTE is insensitive (sensitivity of 55%) compared with TEE for the detection of native valve vegetations [25]. Although others suggest that TTE has a high negative predictive value of 97%, sufficient data are lacking, and further research is needed to better identify those patients with SAB for whom TEE is required to detect endocarditis. [26] The sensitivity of a TEE is 90–100% and is the preferred screening tool in patients with a complicated SAB and suspected infectious endocarditis. [27]

A recent Canadian multicentre study including 847 patients compared the management of SAB in patients receiving infectious disease (ID) consultation and patients without ID consultation of an ID specialist. The number of patients receiving echocardiography increased, but despite bedside consultation of an ID specialist, TEE was only performed in 17% of patients [21]. Others suggested a simple set of criteria for nosocomial SAB to identify patients with low risk of infectious endocarditis and who might not routinely require TEE [28].

A recently published large retrospective 12-year cohort study showed that the use of three evidence-based care processes, appropriate antibiotic therapy, echocardiography and consultation of an ID specialist, was associated with a reduction of 30-day mortality by 67% [29]. The risk-adjusted mortality decreased from 23.5 to 18.2%, lower than in our hospital. In our study, we also found significant effects on adherence to guidelines and the use of echocardiography after the introduction of a protocol. Although execution of our protocol was monitored by a dedicated person (KB), this is not equal to a bedside consultation of an ID specialist.

Despite the improved adherence to guidelines in treating patients with SAB in the post-intervention group, overall mortality did not decrease in our study. Besides the fact that the study itself was not designed to see changes in mortality, the slightly older age and higher incidence of risk factors for complicated SAB in the post-intervention group could be an additional explanation for the absent decrease in mortality. In addition, there was no adjustment for comorbidities and severity of disease performed on admission in both groups. Another possible explanation could be the fact that in this study, the management strategies, including ordering for echocardiography, remained the responsibility of the treating physician. We did not routinely offer bedside ID specialist consultation as was done in other studies of which some reported impressive reductions of mortality [14, 18, 19]. A policy of routine ID consultations and additional thorough search for infectious complications may lead to additional benefits and could be used for future research. However, the lack of improved outcome despite increased quality of care underscores once more that SAB is very difficult to manage, even in the absence of methicillin resistance.

The high mortality related to SAB will probably continue to rise with the increasing use of invasive techniques such as implementation of cardiac devices, prosthetic joints and endovascular prosthesis. In our study, health-care related infections contributed to 50% of all SABs. Tergooi hospital serves a relatively old population, which is reflected by the high age of in-hospital patients with multiple underlying diseases and nosocomial complications.

In conclusion, the management of SAB demands the highest level of clinical commitment. The continuous rise of the use of invasive techniques and devices emphasizes the need for preventive measures and optimal diagnostic and treatment strategies for this high-risk infection. The timely detection of complications of SAB requires more than just clinical expertise. Additional diagnostic techniques are needed. It was recently shown that imaging with F-FDG PET/CT is a valuable technique for early detection of metastatic infectious foci that often leads to modification of treatment and thus reduces mortality. [30]

## Conclusion

Implementation of a hospital-wide protocol for the management of uncomplicated and complicated SAB patients resulted in increased adherence to standard of care. More follow-up blood cultures were obtained, and more echocardiograms were performed. More patients received adequate antibiotic therapy—mostly in the form of an increased duration of therapy. More complicated SAB-patients with infective endocarditis were identified but the overall mortality did not decrease.
