# Correction to: Implementing a hospital-wide protocol for *Staphylococcus aureus* bacteremia

**DOI:** 10.1007/s10096-018-3355-y

**Published:** 2018-08-29

**Authors:** K. Bolhuis, L. J. Bakker, J. T. Keijer, P. J. de Vries

**Affiliations:** 1 Department of Internal Medicine, Academic Medical Center, Meijbergdreef 9, 1105 AZ Amsterdam, The Netherlands; 2 Department of Medical Microbiology, Tergooi Hospital, Van Riebeeckweg 212, 1213 XZ Hilversum, The Netherlands; 3 Department of Cardiology, Tergooi Hospital, Van Riebeeckweg 212, 1213 XZ Hilversum, The Netherlands; 4 Department of Internal Medicine, Tergooi Hospital, Van Riebeeckweg 212, 1213 XZ Hilversum, The Netherlands

**Correction to: European Journal of Clinical Microbiology & Infectious Diseases (2018) 37:1553–1562**

10.1007/s10096-018-3284-9

The article “Implementing a hospital-wide protocol for *Staphylococcus aureus* bacteremia”, written by K. Bolhuis, L. J. Bakker, J. T. Keijer, and P. J. de Vries was originally published electronically on 31 May 2018 with incorrect copyright line in the publisher’s internet portal (currently SpringerLink). The copyright line of the article should be “© The Author(s) 2018”.

